# Towards ultimate low frequency air-core magnetometer sensitivity

**DOI:** 10.1038/s41598-017-02099-z

**Published:** 2017-05-23

**Authors:** Ruben Pellicer-Guridi, Michael W. Vogel, David C. Reutens, Viktor Vegh

**Affiliations:** Centre for Advanced Imaging, The University of Queensland, Brisbane, Queensland Australia

## Abstract

Air-core magnetometers are amongst the most commonly used magnetic field detectors in biomedical instruments. They offer excellent sensitivity, low fabrication complexity and a robust, cost-effective solution. However, air-core magnetometers must be tailored to the specific application to achieve high sensitivity, which can be decisive in the accuracy of the diagnoses and the time required for the examination. Existing methods proposed for the design of air-core magnetometers are based on simplified models and simulations using a reduced number of variables, potentially leading to sensitivity that is suboptimal. To circumvent this we chose a method with fewer assumptions and a larger number of decision variables which employed a genetic algorithm, a global optimisation method. Experimental validation shows that the model is appropriate for the design of highly sensitive air-core magnetometers. Moreover, our results support the suitability of a genetic algorithm for optimization in this context. The new method described herein will be made publicly available via our website to facilitate the development of less costly biomedical instruments using air-core magnetometers with unprecedented sensitivity.

## Introduction

Air-core magnetometers are the preferred choice for many biomedical applications because of their high sensitivity, robustness and inexpensiveness^1^. Although magnetometers with a ferromagnetic core can reach higher sensitivities than air-core magnetometers^2^, they cannot be used in applications where signal or magnetic field distortions are not acceptable. Biomedical applications of air-core magnetometers include magnetic induction tomography (MIT)^3^, ultra-low field magnetic resonance imaging (ULF-MRI)^4^ and magnetocardiography (MCG)^5^. Customizing coil design to specific biomedical applications may lead to a considerable improvement in sensitivity^2^, enabling earlier diagnosis and more accurate monitoring of disease. Additionally, most applications use signal averaging to achieve the desirable signal-to-noise ratio and increasing coil sensitivity would confer the benefit of reduced acquisition time, in proportion to the square of the additional sensitivity.

Air core magnetometer sensitivity is determined by the ratio between the electromotive force (*emf*) induced in the coil and the total electronic noise of the detector. This noise floor of air-core magnetometers is dominated by the thermal noise of the coil, the noise of the pre-amplifier, and the noise of any lumped elements connected to the input of the pre-amplifier, such as tuning capacitors. The task of optimal coil design is that of maximising the ratio between *emf* and all other contributing noise sources. The primary design variables for air core magnetometers are the frequency range, coil size, pre-amplifier properties (gain, noise floor and pre-amplification mode), conductor diameter, and number and location of loops. Some of these variables, such as the outer radius of the coil and its frequency range are delimited often by the application whereas conductor diameter and the number of loops and their locations has to be methodically deduced from a vast range of options.

Various analytical solutions have been proposed to optimise the design of air-core magnetometers^6^ and closely related ferromagnetic-core induction magnetometers^7, 8^. Analytical solutions can offer a direct understanding of how different variables affect the theoretical sensitivity of magnetometers. Furthermore, they provide a function that can be solved for optimal coil variables^9^. A major challenge has been to solve the analytical problem using more accurate and therefore more complex models for the electrical properties of the coil, i.e., resistance, inductance and parasitic capacitance. The accuracy with which these values can be stated directly affects the analytical solution.

Numerical methods have been described for optimising air-core magnetometer designs^5, 10–15^. These have the benefit of requiring fewer assumptions than analytic methods. However, it is very important to match the optimisation algorithm to the problem to mitigate against finding a local instead of a global solution^9^. Estola *et al*. simulated a number of magneto-cardiograms with a non-rectangular coil cross-section for detecting a near field source in the band 0.5–100 Hz^5^. Wire diameter was determined using a brute force strategy with simulated designs based on a simplified model for the magnetic source and by employing an established computer aided design methodology. Chen *et al*. described an optimisation method which maximised signal-to-noise ratio (SNR) and minimised coil diameter for coils operating in the range of tens of Hz to 71 kHz^10^. Here, the coil design was limited to a single layer and a particular weight, and the search for the optimal number and diameter of coil loops was deduced from a plot of sensitivities for different coil layouts. An optimal broadband air-core magnetometer, based on the Brooks coil, was later developed and its sensitivity was analysed in the absence of amplifier noise^16^. Subsequent work yielded the optimised minimum air-core coil size based on a fixed cut-off frequency and a specific type of amplifier^12^. Details about the optimisation algorithm used to obtain the variables was, however, not provided. An air-core magnetometer has also been designed specifically for ultra-low-filed magnetic resonance imaging instruments^11^. The average coil diameter was optimised and conductor thickness was chosen based on skin depth for copper wire tuned to 3 kHz. Additional work on ferromagnetic-core magnetometers has focused on maximising sensitivity with constraints on the weight and size of the coils^14, 15^.

Our approach to designing air-core magnetometers is to expand the search space of the optimisation process with the objective of increasing coil sensitivity. To achieve this goal, we use more accurate but more complex analytical expressions, the variables of which are deduced using a global optimisation procedure. We allow conductor diameter, distance between wires, number of coil layers and number of turns per layer to be free variables. A genetic algorithm is employed to search for the globally optimal solution. In what follows, numerical models for two popular pre-amplification modes are first presented, followed by an outline of non-tuned current-to-voltage and tuned voltage-to-voltage designs. The non-tuned current-to-voltage design, commonly known as the trans-impedance amplifier, is desirable in many applications due to the linear frequency gain response and excellent sensitivity at very low frequencies^5–8, 10, 12, 14^. The tuned voltage-to-voltage design has the potential to provide additional sensitivity with narrower bandwidths, most often used in high frequency applications. Empirical measurements are used to validate the numerical models employed in the optimisation of air-core magnetometers.

## Methods

### Numerical model

#### Coil

**Figure 1:**
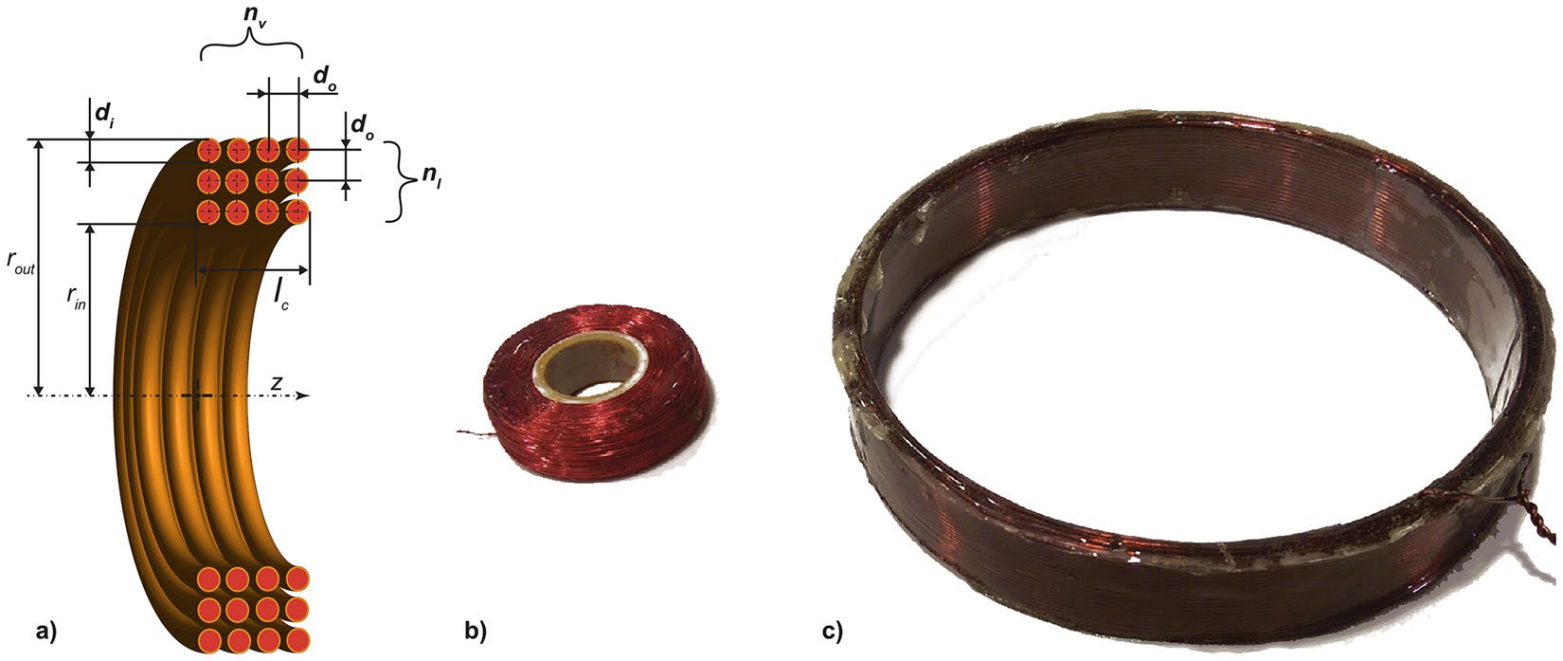
Cross-section of the coil and picture of built coil prototypes. (**a**) Optimisation variables are shown in bold: d_i_ is the conductor diameter, d_o_ is the conductor spacing, n_l_ the number of layers and n_v_ the number of loops per layer. Inner and outer radius of the coil are represented as r_in_ and r_out_, respectively. (**b**) The small coil comprises 38 layers, 43 loops per layer with an outer radius of 19 mm and height of 10 mm. The copper conductor has a diameter of 0.2 mm and conductors are spaced 0.22 mm apart. (**c**) The big coil comprises 5 layers, 21 loops per layer with an outer radius of 60 mm and height of 18 mm. The copper conductor has a diameter of 0.8 mm and conductors are spaced 0.86 mm apart.

The electrical properties of the coil are represented by the AC resistance (*R*_*S_AC*_), inductance (*L*_*S*_) and the parasitic capacitance (*C*_*S*_) based on knowledge of conductor location and diameter. Assuming wires are equally distributed within the coil winding, individual locations are estimated based on coil outer diameter, number of layers and turns per layer, conductor diameter and wire spacing (see Fig. 1a). In the case of a single strand solid conductor the AC resistance can be calculated as^17^:

1 $${R}_{S\_AC}={R}_{S\_DC}(1+F(z)+u(N)\frac{{d}_{i}^{2}}{{d}_{o}^{2}}G(z)),$$

2 $$F(z)=\frac{{z}^{2}}{8}Im\frac{{J}_{3}(z\sqrt{-i})}{{J}_{1}(z\sqrt{-i})},\,G(z)=\frac{{z}^{2}}{8}Im\frac{{J}_{2}(z\sqrt{-i})}{{J}_{0}(z\sqrt{-i})},$$

3 $$z=\sqrt{\frac{{d}_{i}^{2}}{4}\omega {\mu }_{0}/{\rho }_{copper}},$$

where *F(z)* and *G(z)* are functions representing the skin depth and conductor proximity effects, respectively, and *J* corresponds to Bessel functions of the first kind. *u(N)* depends on the separation of conductor centres (*d*_*o*_ in Fig. 1a), radius of individual loops (*r*_*loop*_), z-axis offset of each loop (*z*_*loop*_) and the number of loops (*N*). Thereby,

4 $$u(N)=\frac{1}{N}{\sum }_{i=1}^{N}{(\sum _{j=1,j\ne i}^{N}\frac{{r}_{loop,j}-{r}_{loop,i}}{{({r}_{loop,j}-{r}_{loop,i})}^{2}+{({z}_{loop,j}-{z}_{loop,i})}^{2}})}^{2}+{(\sum _{j=1,j\ne i}^{N}\frac{{z}_{loop,j}-{z}_{loop,i}}{{({r}_{loop,j}-{r}_{loop,i})}^{2}+{({z}_{loop,j}-{z}_{loop,i})}^{2}})}^{2}.$$

In the case of Litz wires, the calculation of *R*_*S_DC*_ and *R*_*S_AC*_ is obtained via equations (5, 6) with *t*_*yarn*_ being the outer insulation of the wire, and with *N*_*S*_, *N*_*B*_ and *N*_*C*_ defining the number of strands, number of bunching and cabling operations of the Litz wire respectively. The packing factor *p* is predefined: *p* = 1.25 for *N*_*S*_ 1 ≤ 1; *p* = 1.26 for 11 < *N*_*S*_ ≤ 15; *p* = 1.27 for 15 < *N*_*S*_ ≤ 24; and *p* = 1.26 for 24 < *N*_*S*_ < 400. Hence,

5 $${R}_{S\_AC}={R}_{S\_DC}(1+F(z)+(u(N)+2)\frac{{N}_{S}^{2}{d}_{i}^{2}}{{d}_{o}^{2}}G(z)),$$

6 $${R}_{S\_DC}=\,{R}_{S\_DC\_strand}\frac{{1.015}^{{N}_{B}}{1.025}^{{N}_{C}}}{{N}_{S}};{d}_{i\_strand}=\frac{{d}_{i}-{t}_{yarn}}{p\sqrt{{N}_{S}}}.$$

Coil inductance and capacitance are calculated numerically using a previously described method^18^. The total inductance is a sum of self-inductance (*L*_*0*_) and mutual inductance (*M*_*0*_) such that *L*_*S*_ = *L*_*0*_ + *M*_*0*_. The self-inductance employs the argument, *k* ^*2*^_*Lo*,*i*_ = *4r*_*loop*,*i*_∙(*r*_*i*_ − *d*_*i*_/*2*)/(*2r*_*i*_ − *d*_*i*_ /*2*)^2^, where *μ*_*0*_ is the magnetic permeability of air, and *E* and *K* are elliptic integrals of first and second kind, such that

7 $${L}_{0}={\mu }_{0}\sum _{i=1}^{N}(2{r}_{loop,i}-\frac{{d}_{i}}{2})[(1-\frac{1}{2}{k}_{Lo,i}^{2})K({k}_{Lo,i})-E({k}_{Lo,i})].$$

As for *M*_*0*_, the arguments of *k*_*Mo*_ incorporate an extra dimension to capture the mutual coupling between conductors, hence *k* ^*2*^_*Mo,i,j*_ = *4r*_*loop,i*_∙*r*_*loop,j*_ */*((*r*_*loop,i*_ + *r*_*loop,j*_)^*2*^ + (*z*_*loop,i*_ *− z*_*loop,j*_)^2^). The mutual inductance is

8 $${M}_{0}=2{\mu }_{0}\sum _{i=1}^{N}\sum _{j=1,j\ne i}^{N}\frac{\sqrt{{r}_{loop,i}-{r}_{loop,j}}}{{k}_{i,j}}[(1-\frac{1}{2}{k}_{Mo,i,j}^{2})K({k}_{Mo,i,j})-E({k}_{Mo,i,j})].$$

The equation for stray capacitance

9 $${C}_{s}=\frac{8\pi {\varepsilon }_{0}{\varepsilon }_{r}{l}_{c}({n}_{l}-1)}{6{{N}_{L}}^{2}(1.26{d}_{o}-1.15{d}_{i})}[2{r}_{in}+{d}_{o}]$$

was proposed by Martinez *et al*.^18^, and is expressed here for the case of equally spaced conductors. Equation (9) is a function of the relative electrical permittivity of the coating layer (*ε*_*r*_), coil length (*l*_*c*_), number of layers (*n*_*l*_) and coil internal radius *r*_*in*_, and *ε*_*0*_ is the electrical permittivity of free-space. *emf* induced in the coil per Tesla is calculated by *emf* = *j2πf* ∑^*N*^_*i*=*1*_ *πr* ^*2*^_*loop,i*_.

#### Non-tuned current-to-voltage amplifier design

**Figure 2:**
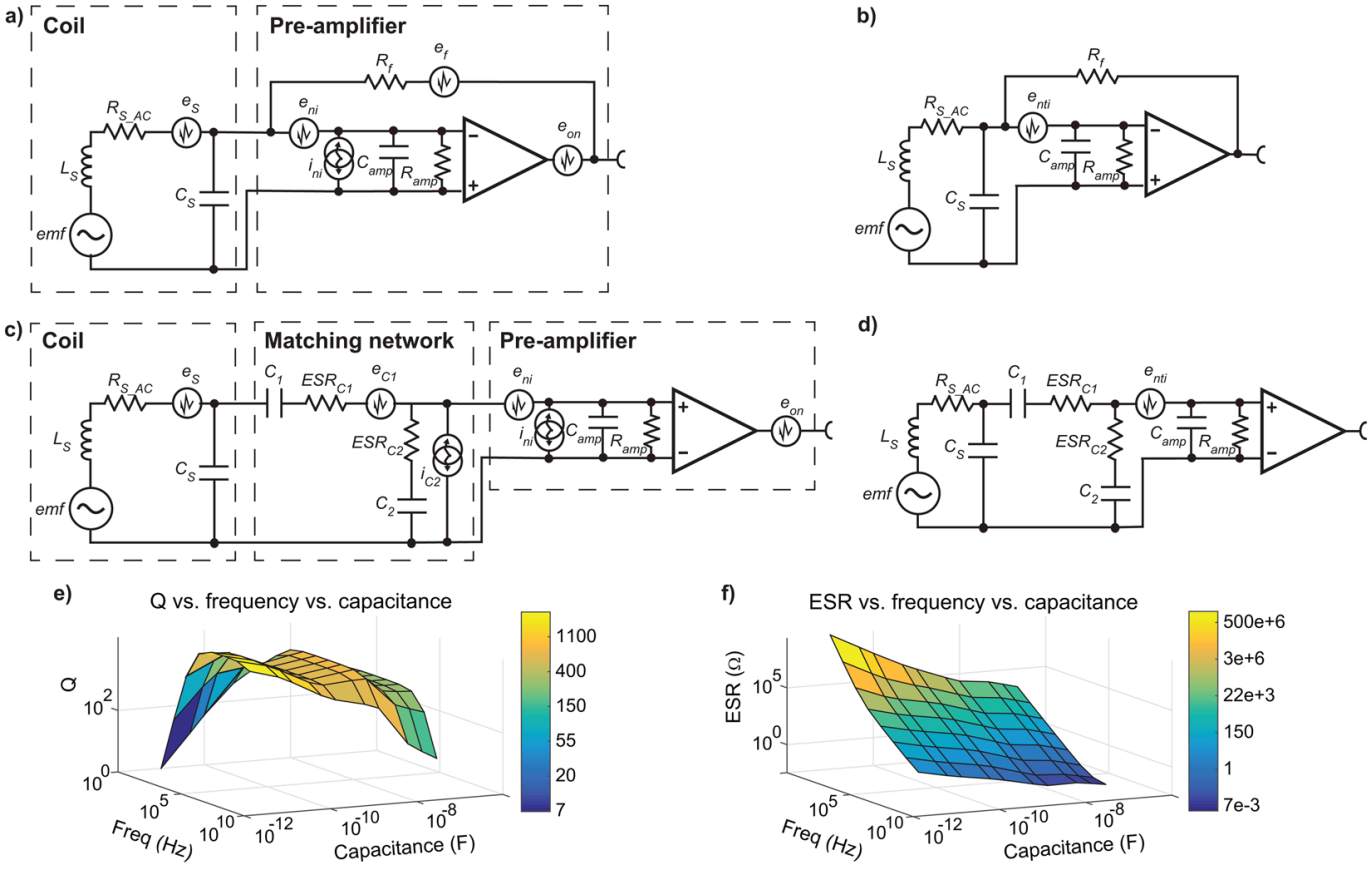
Schematics of considered pre-amplification modes and plots of the Q and the ESR of the look-up table employed for the tuning and matching capacitors. The equivalent circuit of the coil and pre-amplifier for the non-tuned current-to-voltage depicting (**a**) the individual noise sources, and (**b**) the equivalent total voltage noise, e_nti_. Shown are individual equivalent circuits for coil, matching network and pre-amplifier for the tuned voltage-to-voltage pre-amplification elucidating (**c**) the individual noise sources, and (**d**) the equivalent total voltage noise. (**e**) Plots the interpolated look-up table employed to estimate the quality factor, Q, of tuning and matching capacitors, and (**f**) plots its respective equivalent series resistance, ESR. Note the high dependency of Q and ESR on capacitance and frequency of operation.

In this design shown in Figure 2a the electric current generated in the coil is measured. The noise floor of the setup is used to estimate magnetometer sensitivity (*ζ*) such that

10 $$\zeta (f)=emf(f)/{e}_{nti}(f).$$

Here, *e*_*nti*_(*f*) represents the frequency dependent total equivalent voltage noise at the input of the pre-amplifier (v/√Hz) schematically shown in Fig. 2b. This total noise is calculated from all the individual noise sources depicted in Fig. 2a. They are treated as a voltage noise source at the input of the pre-amplifier in series with the voltage noise of the pre-amplifier *e*_*ni*_, in such a way they can be added as *e* ^*2*^_*nti =*_ *e* ^*2*^_*ni*_ + *e* ^*2*^_*ii*_ + *e* ^*2*^_*si*_ + *e* ^*2*^_*fi*_ + *e* ^*2*^_*oni*_. Noise sources at the input of the amplifier are

11 $${e}_{ii}={i}_{in}|{Z}_{s}|,$$

12 $${e}_{si}=\sqrt{4{k}_{b}T{R}_{s}},$$

13 $${e}_{oni}=\frac{{e}_{on}}{G};G=\frac{|{R}_{f}+{Z}_{s}|}{|{Z}_{s}|},$$

14 $${e}_{fi}=\frac{{e}_{f}}{G};{e}_{f}=\sqrt{4{k}_{b}T{R}_{f}},$$

15 $${X}_{Ls}=j2\pi f{L}_{s},$$

16 $${X}_{Cs}=\frac{1}{j2\pi f{C}_{s}},$$

17 $${Z}_{s}=\frac{{X}_{Cs}({R}_{s}+{X}_{Ls})}{{R}_{s}+{X}_{Ls}+{X}_{Cs}},$$

where *Z*_*S*_ is coil impedance, *k*_*b*_ is Boltzmann’s constant (1.38e-23 J/K) and *T* is the temperature (K). *e*_*ii*_ represents the equivalent voltage noise at the input of the pre-amplifier arising from the input current noise, *i*_*in*_, of the pre-amplifier. *e*_*si*_ and *e*_*f*_ are the thermal voltage noises produced by the coil and the feedback resistor *R*_*f*_, respectively. *e*_*on*_ is the minimum voltage noise at the output of the pre-amplifier provided by the manufacturer. We assume current and voltage noise sources to be uncorrelated.

#### Tuned voltage-to-voltage amplifier design

Tuned magnetometers use a resonant circuit to enhance the sensitivity at a specific frequency of operation. We choose the inverted-L matching network configuration shown in Fig. 2c and d since it provides wider bandwidth than the T or pi networks, uses a small number of capacitors and does not incorporate extra inductors which may lead to additional signal losses especially at low frequencies. The loss introduced by the parasitic resistance of the capacitors is calculated through their equivalent series resistance ESR = 1/2π*fCQ*, being *Q* the quality factor and *C* the capacitance.

The tuning and matching network is necessary to set the frequency (*f*_*0*_) of the coil as previously described^19^. At this frequency, the tuning and matching network transforms the coil impedance (*Z*_*S*_) to the apparent source impedance (*R*_*match*_) measured at the input of the amplifier, and the transformation ratio is *m* = *R*_*match*_ /*R*_*S_AC*_. For this transformation, the required quality factor *Q*_*req*_ of the resonant circuit comprising the coil and the tuning and matching network is first calculated using *Q*_*req*_ = √(*m*−1). Then, the total reactance is obtained (*X*_*S_total*_ = *Q*_*req*_ *∙R*_*S*_). Additionally, the reactance of the series network capacitor *C*_*1*_ is computed (*X*_*s_tun*1_ = *X*_*S_total*_ − *X*_*S*_, where *X*_*S*_ is the imaginary part of *Z*_*S*_). Finally, the equivalent parallel impedance, the conjugate of the impedance of the second tuning capacitor (*C*_*2*_), is calculated (*X*_*S_total_p*_ = X_*S_total*_∙(1 + 1/*Q* ^2^_*req*_)). The input capacitance of the amplifier (*C*_*amp*_) is also considered. The values for the tuning capacitors are:

18 $${C}_{1}=\frac{1}{j2\pi {f}_{0}({X}_{S\_tun1})},$$

19 $${C}_{2}=\frac{1}{j2\pi {f}_{0}-({X}_{S\_total\_p})}-{C}_{amp}.$$

The ESR of the capacitors is determined using a look-up table created from their corresponding datasheets. Both *Q* and ESR are dependent on the operating frequency and capacitance as shown in Fig. 2e and f.

The effective gain (*G*_*eff*_) the *emf* experiences has to account for losses due to the incorporation of the matching network capacitors. Additionally, thermal noise sources are added for each of the capacitors (*e*_*C1*_ for *ESR*_*C1*_ and *e*_*C2*_ for *ESR*_*C2*_). The total voltage noise is then defined as *e* ^2^_*nti*_ = *e* ^2^_*ni*_ + *e* ^2^_*ii*_ + *e* ^2^_*si*_ + *e* ^2^_*C1i*_ + *e* ^2^_*C2i*_ + *e* ^2^_*oni*_ measured at the amplifier input. The sensitivity can be stated as

20 $$\zeta (f)=emf(f){G}_{eff}(f)/{e}_{nti}(f);$$

*G* is the gain of the pre-amplifier in equation (25). In summary, the values for the individual components of the tuned voltage-voltage design can be calculated as:

21 $${e}_{ii}={i}_{n}|{Z}_{th}|,$$

22 $${e}_{si}=|{G}_{eff}|\sqrt{4{k}_{b}T{R}_{s}},$$

23 $${e}_{C1i}=|{G}_{C1}|\sqrt{4{k}_{b}T\cdot ES{R}_{C1},}$$

24 $${e}_{C2i}={i}_{C2i}|{Z}_{th}|;{i}_{C2i}=\sqrt{\frac{4{k}_{b}T}{EP{R}_{C2}},}$$

25 $${e}_{oni}=\frac{{e}_{on}}{G},$$

26 $${Z}_{th}=\frac{{Z}_{a\_C1}({Z}_{S}+{X}_{C1}+ES{R}_{C1})}{{Z}_{a\_C1}+{Z}_{S}+{X}_{C1}+ES{R}_{C1}},$$

27 $$EP{R}_{C2}=ES{R}_{C2}[1+{(\frac{1}{2\pi f{C}_{2}ES{R}_{C2}})}^{2}],$$

28 $${Z}_{a\_C1}=\frac{1}{\frac{1}{{X}_{C2}+ES{R}_{C2}}+\frac{1}{{X}_{Camp}}+\frac{1}{{R}_{amp}}},$$

29 $$Z{\text{'}}_{a\_C1}=\frac{{X}_{Cs}({Z}_{a\_C1}+{X}_{C1}+ES{R}_{C2})}{{X}_{Cs}+{Z}_{a\_C1}+{X}_{C1}+ES{R}_{C2}},$$

30 $${G}_{eff}=\frac{Z{\text{'}}_{a\_C1}}{Z{\text{'}}_{a\_C1}+{X}_{Ls}+{R}_{s}},$$

31 $${G}_{C1}=\frac{{Z}_{a\_C1}}{{Z}_{a\_C1}+{Z}_{S}+{X}_{C1}+ES{R}_{C1}}.$$

#### Validation of numerical models

A small and a large coil (Fig. 1b) are built along with non-tuned and tuned pre-amplifiers which employ the instrumentation amplifier INA217. The small coil uses 38 layers with 41 loops per layer, resulting in an outer radius of 19 mm and height of 10 mm. The copper conductor has a diameter of 0.2 mm and conductors are spaced 0.24 mm apart. The bigger coil has 5 layers with 21 loops per layer, resulting in an outer radius of 60 mm and height of 18 mm. The copper conductor in this case has a 0.8 mm diameter and is spaced 0.86 mm apart.

Coil sensitivity is determined using a combination of two measurements. First, the magnetometer is located coaxially in the centre of a Helmholtz pair and the field is calculated as *B* = 8*μ*_*0*_ *N*_*Helm*_ *I*/(5√5*a*), where *μ*_*0*_ is free-space permeability (4π10^−7^ T·m/A), *I* is the current in the coils, *a* is coil radius and *N*_*Helm*_ is the number of turns in each Helmholtz coil. Second, the electronic noise floor of the magnetometer is measured by placing the magnetometer in a portable magnetically shielded box. The shielded box is made out of 8 layers of 0.5 mm thick electrical steel, and its dimensions are 1 m × 1 m × 1.5 m. Noise floor measurements are performed with the box in two different locations having at least one order of magnitude difference in environmental noise. This is to verify that sensitivity estimation of the magnetometer is not being affected by environmental electromagnetic noise. The noise floor measurement of the magnetometers did not change with the location of the shielded box, with the exception of the most sensitive coil, i.e., the big coil in the tuned configuration. For this coil the average noise floor reduced by 18% with the shielded box in the location with less electromagnetic radiation. This confirms that the sensitivity estimations conducted in the location with less environmental noise are reliable. The sensitivity of the magnetometer (T/√Hz) is then calculated by dividing the measured noise floor (V/√Hz) by the measured field to voltage conversion ratio (V/T).

#### Optimisation algorithm

The optimisation algorithm allows the following decision variables to evolve: number of layers, number of loops per layer, and conductor diameter and spacing. Other variables, such as coil outer diameter, amplifier noise sources and ESR look-up table for the capacitors are user defined.

The search strategy for noise matching is performed differently for the case of tuned and the non-tuned configurations. In the case of the non-tuned design, the genetic algorithm searches for the optimum noise configuration because it has control on the impedance of the coil. In the case of the tuned design, the transformation ratio of the matching network is adjusted by selecting the best sensitivity over a range of conversion ratios for each coil configuration. *R*_*match*_ values are limited to the range *e*_*ni*_/*i*_*ni*_ ≤ *R*_*match*_ ≤ 4*k*_*b*_ *T*/*i*_*n*_^2^, the upper limit corresponds to where amplifier *e*_*ii*_ matches coil *e*_*si*_.

To find optimal values for the decision variables, ga, an inbuilt MATLAB® function is used. Given that the decision variables “number of layers” and “number of loops per layer” are integer-valued, the algorithm incorporates special creation, crossover, and mutation functions as described in^20^. This genetic algorithm attempts to minimise a penalty function, which is formulated from the fitness function of feasible population members plus a penalisation comprising constraint violations^21^. Equation (10) is used as the cost function for the non-tuned case, and equation (20) for the tuned case. We use the following bounds: 1 < *n*_*v*_ < 100, 1 < *n*_*l*_ < 100, 0.101 < *d*_*i*_ < 5 (mm) and 0.1 < *d*_*o*_ < 4.9 (mm) and constraint: *d*_*i*_ < *d*_*o*_. A penalty is imposed to prevent solutions in which the self-resonant frequency of the coil (*f*_*self*_) is close (*f*_*self*_ ≤ 10*f*_*o*_) to the frequencies of interest (*f*_*0*_). Similarly, a penalty forces the gain *G* of Equation (13) to be smaller than the differential gain of the amplifier. Simulations are executed on an eight core Intel computer with an i7-2600 3.4 GHz CPU and 16 GB RAM.

Reproducibility is assessed for a number of different magnetometer setups and computational times are recorded. Sensitivity curves for different configurations are analysed. The influence of the matching and tuning network, of lossy capacitors and of using Litz wires are analysed as well. The optimal solutions obtained with the proposed method are compared to the Brooks coil design.

## Results

We first validate the numerical models against experimental measurements performed in two coils for the non-tuned current-to-voltage and tuned voltage-to-voltage amplification configurations. Afterwards we evaluate the optimisation algorithm.

### Numerical model

Table 1 quantifies the error between predicted and measured electrical properties of the in-house built air-core magnetometers. The simulated and measured DC resistance and AC resistance at 10 kHz, inductance, and capacitance of prototypes differed by only 2%, 3%, 4% and 15%, respectively.

**Table 1:** Comparison of the electrical properties between simulated and empirically measured values in two different in-house built coils.

	Coil param. (u. & mm)					*R*_*S_DC*_ (Ω)		*R*_*S_AC*_ (Ω) 10 kHz		Induct. (mH)		Paras. Cap. (pF)	
	n_l_	n_v_	d_i_	d_o_	r_out_	Theo.	Meas.	Theo.	Meas.	Theo.	Meas.	Theo.	Meas.
Small coil	38	41	0.2	0.24	19	71.2	72.4	75.1	76.4	57.4	55.5	18	21
Big coil	5	21	0.8	0.86	60	1.29	1.31	2.14	2.2	2.15	2.16	258	261

Variables are number of layers (n_l_), number of loops per layer (n_v_), conductor diameter (d_i_) and spacing (d_o_), coil outer radius (r_out_), DC resistance (R_S_DC_), AC resistance at 10 kHz (R_S_AC_), inductance and parasitic capacitance.

**Figure 3:**
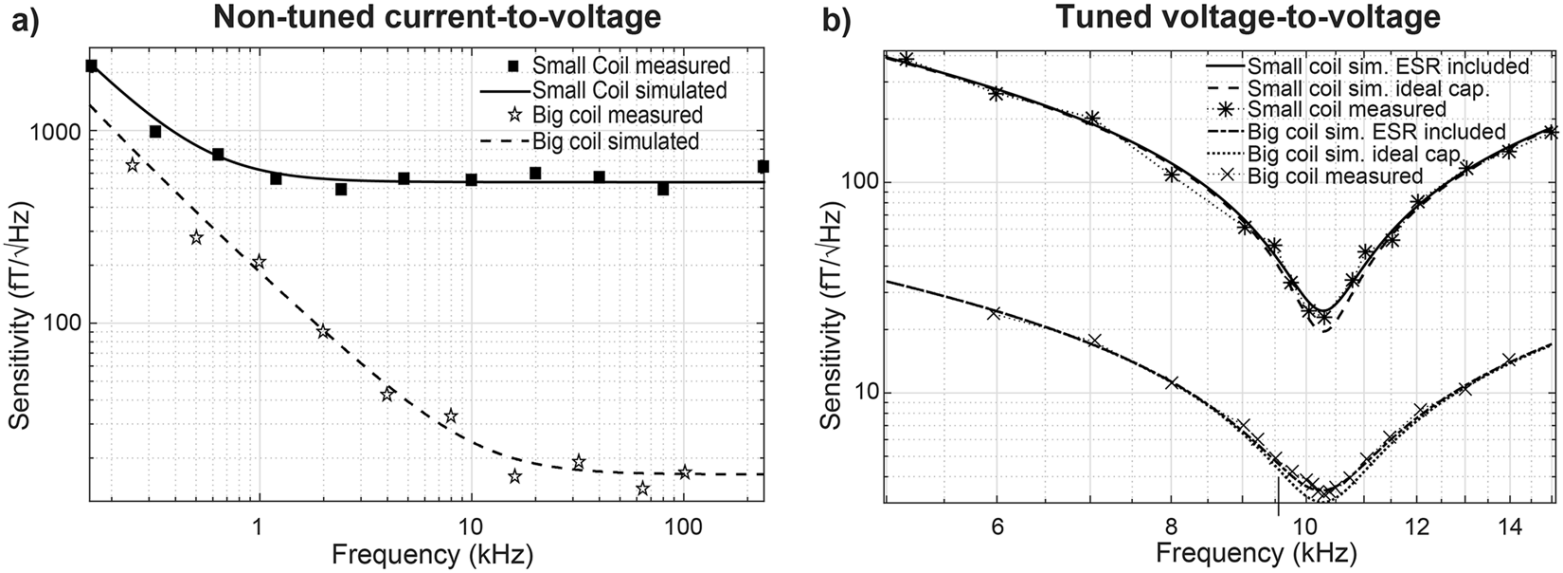
Sensitivity of prototype coils. Sensitivity comparison between measured and estimated values for (**a**) non-tuned current-to-voltage and (**b**) tuned voltage-to-voltage with ideal and lossy capacitors.

The magnetic field sensitivity of both coils for current-to-voltage and voltage-to-voltage configurations is provided in Fig. 3. The non-tuned current-to-voltage coil sensitivity in the range 1 kHz − 100 kHz is shown in Fig. 3a. The simulated and measured mean sensitivities are 544 fT/√Hz and 558 fT/√Hz for the small coil and 22.7 fT/√Hz and 22.3 fT/√Hz for the large coil, respectively. Figure 3b shows the sensitivities for the same coils but with tuned voltage-to-voltage amplification at 10 kHz. For a 1 kHz bandwidth, the simulated and measured sensitivities for the small coil are 24.5 fT/√Hz and 23.7 fT/√Hz and simulation with ideal capacitor yields a sensitivity of 19.6 fT/√Hz. Corresponding peak sensitivities are 29.7 fT/√Hz, 30.3 fT/√Hz and 26.2 fT/√Hz. Similarly, for the lager coil the mean values are 3.9 fT/√Hz for measured, 3.8 fT/√Hz simulated with lossy capacitors and 3.4 fT/√Hz with ideal capacitors. Peak sensitivities are 3.3 fT/√Hz, 3.4 fT/√Hz and 3.2 fT/√Hz. By accounting for ESR in the simulations, the accuracy of the numerical model improves by 80% for the small coil and by 75% for the big coil.

### Optimisation algorithm

The reproducibility of the optimisation procedure is evaluated by rerunning the algorithm for a specific case (*r*_*out*_ = 45 mm, *f*_*0*_ = 10 kHz, *bw* = 1 kHz, amplifier INA127, *Q*_*C1*_ ≈ *Q*_*C2*_ ≈ 200) resulting in a coefficient of variation of 1.5% for non-tuned current-to-voltage magnetometers and 0.6% for the tuned voltage-to-voltage configuration. The average optimal sensitivity is 2.4% higher than the best solution found by the solver in the non-tuned case and 0.5% in the tuned mode. The time to reach a solution scales with the number of coil loops in the design ranging from one minute to optimise a 300 loop coil configuration up to as long as 60 minutes for a 5000 loop coil arrangement.

**Figure 4:**
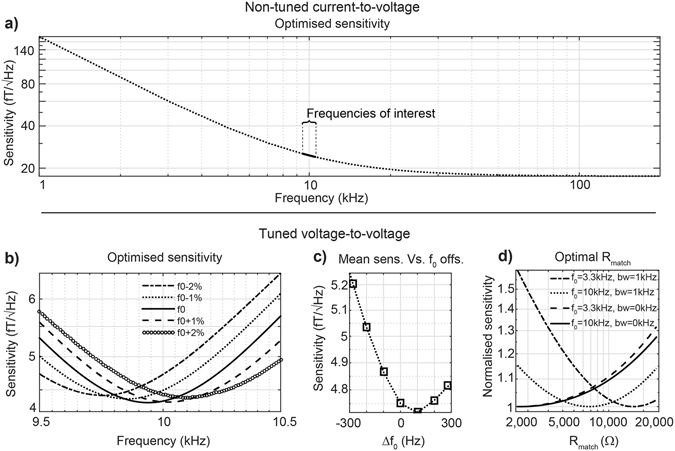
Plots of optimised sensitivity profiles and effects of tuning frequency offsets and matching impedance in average sensitivity. (**a**) Sensitivity vs. frequency curve of a non-tuned current-to-voltage amplifier, indicating the frequencies for optimal sensitivity operation. (**b**) Sensitivity for a coil optimised for 9.5–10.5 kHz bandwidth tuned to 9.8, 9.9, 10, 10.1, and 10.2 kHz and the (**c**) effect of these tuning frequencies on average sensitivity. (**d**) Normalised mean sensitivity as a function of different equivalent resistances R_match_ for two different centre frequencies (3.3 and 10 kHz) and bandwidths (0 and 1 kHz) in tuned voltage-to-voltage coils.

The relationship between sensitivity and frequency differs between both pre-amplification configurations. For the non-tuned current-to-voltage configuration, the optimal sensitivity range is found at the point in the sensitivity plot where the negative slope changes from a steep to a milder descent as can be seen in Fig. 4a. In the bandwidth of interest, the sensitivity improves with increasing frequency. The tuned case has the best sensitivity in the vicinity of the resonant frequency. The corresponding sensitivity peak is shown in Fig. 4b. Notably, the fact that the sensitivity peak is in the centre of the bandwidth does not necessarily mean optimal sensitivity. This is corroborated in Fig. 4c, where the mean sensitivity of a set of coils optimised for a bandwidth fixed between 9.5 kHz and 10.5 kHz with different offsets in the tuning frequency (*r*_*out*_ = 45 mm, *f*_*0*_ = 10 kHz, *bw* = 1 kHz, INA127, *Q*_*C1*_ ≈ *Q*_*C2*_ ≈ 200), is shown. The horizontal axis represents the offset of the tuning frequency with respect to the centre frequency of 10 kHz. Here, the sensitivity improvement between tuning to the centre frequency or to an optimal frequency offset of +100 Hz is less than 1%.

Optimised equivalent coil resistance at the centre frequency measured at the amplifier input (*R*_*match*_) is within proposed search space for four cases evaluated (*r*_*out*_ = 45 mm, amplifier INA127, *Q*_*C1*_ ≈ *Q*_*C2*_ ≈ 200), all of which are shown in Fig. 4d. In two of the cases (solid line and broken line) the bandwidth (*bw*) is set to 0 Hz, that is, the coil is optimised to give the best performance at a single frequency, and the optimal solution lies near the classical value of *R*_*match_class*_ = *e*_*n*_/*i*_*n*_. However, setting the bandwidth to 1 kHz shifts the optimal *R*_*match*_ value higher (dotted line and dot-slash line). This improves sensitivity by 16% for *f*_*0*_ = 10 kHz and 60% for *f*_*0*_ = 3.3 kHz with respect to *R*_*match_class*_.

**Figure 5:**
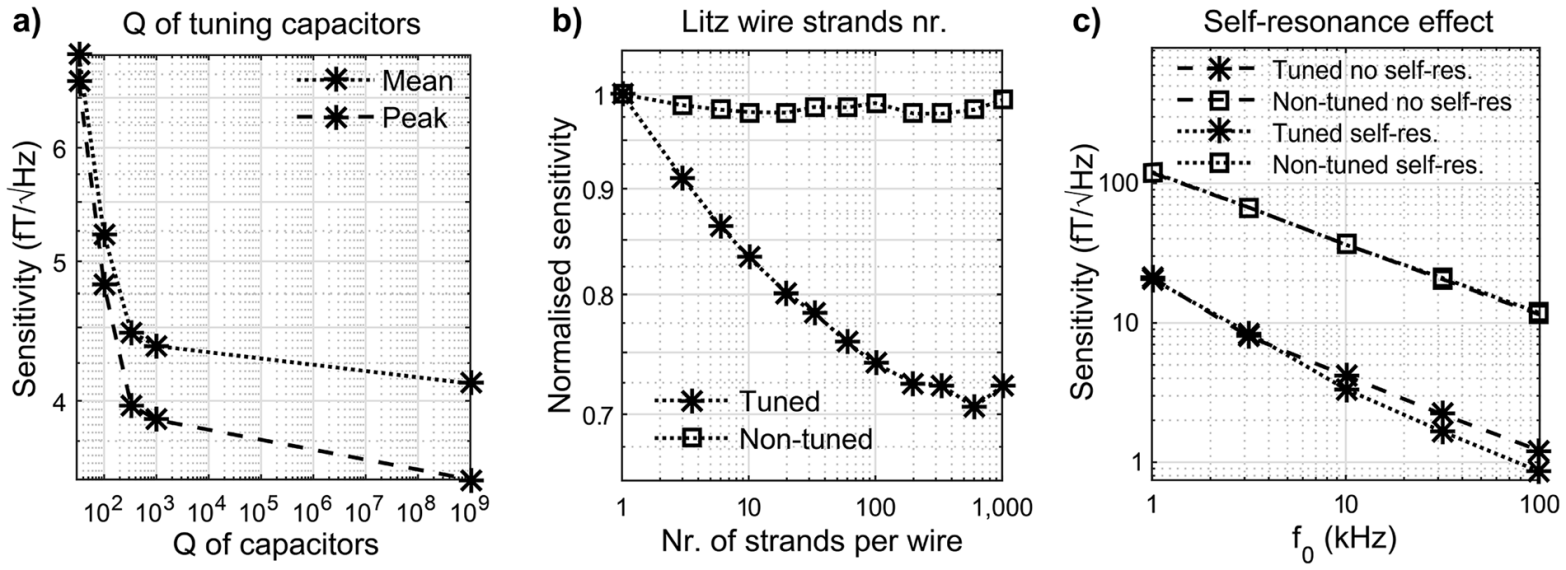
Enhancement of sensitivity. (**a**) Effect of the quality factor of the capacitors on the mean and peak sensitivities of the tuned voltage-to-voltage air-core magnetometer. (**b**) Normalised sensitivity as a function of number of strands per wire illustrated for the non-tuned current-to-voltage (squares) and tuned voltage-to-voltage (asterisks) configurations. (**c**) Comparison of the solutions from the optimisation algorithm with (broken lines) and without (dotted lines) self-resonant frequency penalisation.

The simulation result shown in Fig. 5a indicates that high quality factor capacitors have a significant effect on sensitivity. For the tuned voltage-to-voltage configuration (*r*_*out*_ = 45 mm, *f*_*0*_ = 10 kH, *bw* = 1 kHz, amplifier INA217) with low quality capacitors (*Q* = 33), the average sensitivity is decreased by as much as 69% compared to the sensitivity with higher quality capacitors (*Q* = 1000). Notably, Fig. 5a provides shows this effect having greatest impact below a quality factor of ~330. Beyond this level of capacitor *Q* the improvement is reduced. Not unexpectedly, the effect of higher *Q* is greater on peak sensitivity than on mean sensitivity.

Considering the non-tuned current-to-voltage and tuned voltage-to-voltage configurations using the LNA718 amplifier (*r*_*out*_ = 45 mm, *l*_*max*_ = 20 mm, *f*_*0*_ = 100 kHz, *bw* = 10 kHz, *Q*_*C*_ ≈ *Q*_*C2*_ ≈ 200), Fig. 5b shows the effect of increasing the number of strands per wire. Results have been normalised against the case of a single strand wire. The use of a Litz wire does not appear to provide great benefits for the non-tuned case. For the tuned configuration, an improvement in sensitivity of around 39% can be achieved with around 500 strands per wire, while for the non-tuned case, a maximum of 2% improvement is achieved with 200 strands per wire. It is worth noting that, unlike in the non-tuned case, thermal noise from the coil is dominant in the tuned setup.

We evaluated how much sensitivity can be gained through the self-resonance effect by examining the effect of removing penalisation with respect to the self-resonance frequency of the coil in the optimisation algorithm. Figure 5c shows results for an example configuration (amplifier INA217, *r*_*out*_ = 45 mm, *l*_*max*_ = 20 mm, *bw* = 0 Hz, *Q*_*C1*_ ≈ *Q*_*C2*_ ≈ 200) for both the non-tuned and tuned configurations. An effect cannot be observed for the non-tuned configuration, whilst more than 30% increase in sensitivity is predicted above 10 kHz for the tuned configuration.

**Figure 6:**
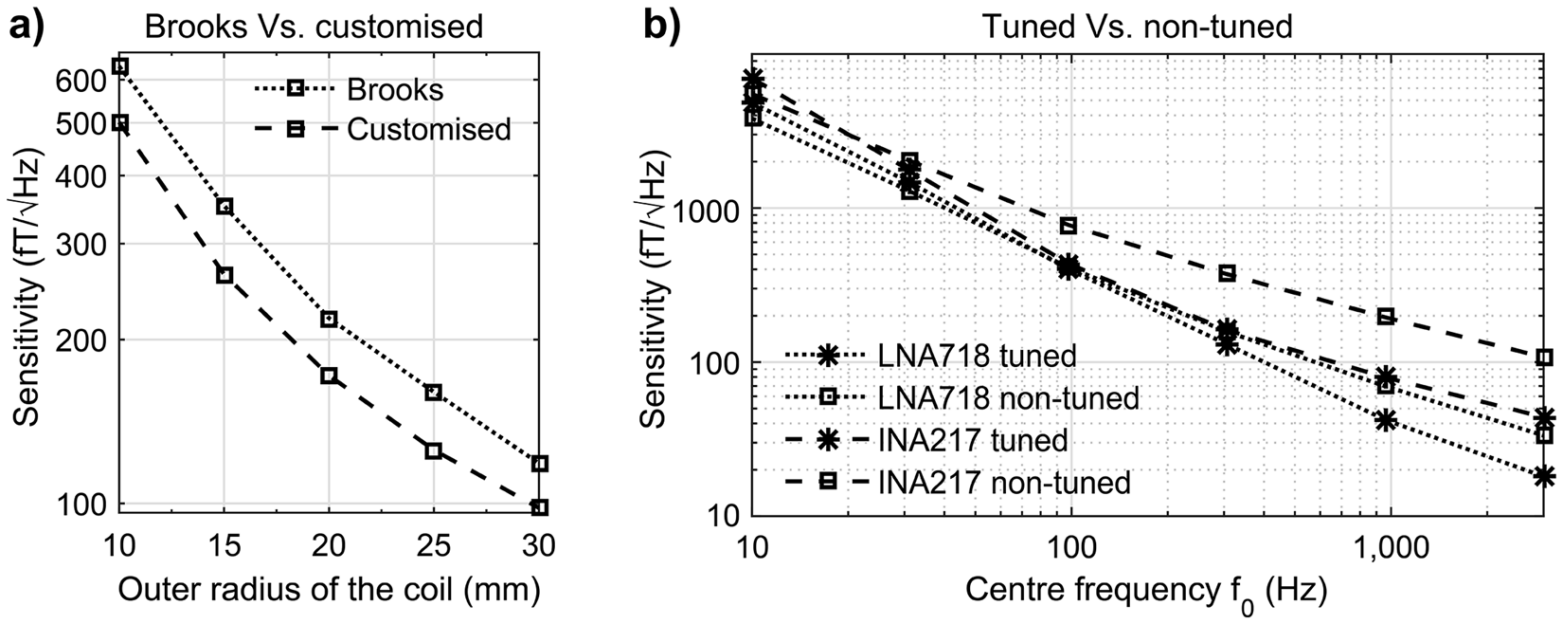
Sensitivity performance comparison. (**a**) Mean sensitivity achieved by a Brooks coil (square cross section) with non-tuned current-to-voltage amplification compared with rectangular cross section non-tuned current-to-voltage configuration. (**b**) Mean sensitivity comparison in between optimised non-tuned current-to-voltage (squares) and tuned voltage-to-voltage (asterisks) amplification with the pre-amplifiers LNA718 (broken lines) and INA217 (dotted lines).

Brooks coils have a particular shape consisting of a square cross section with an outer radius twice the coil height. It has been suggested that this design maximises the sensitivity of non-tuned current-to-voltage configuration by maximising the ratio of inductance to resistance^12^. In Fig. 6a we compare the sensitivities for Brooks coils versus our proposed customised design method. The outer radius is constrained to be the same for both coils, but the height and inner radius is allowed to vary in the customised design. Optimised Brooks coils have 24 × 24, 19 × 19, 20 × 20, 17 × 17 and 18 × 18 turns, while the customised algorithm finds 50 × 10, 50 × 8, 48 × 7, 47 × 6 and 44 × 6 to be the optimum turns per layer and number of layers for coils with outer radii of 10, 15, 20, 25 and 30 mm respectively. The corresponding inductance to resistance ratio are 7.1146e-04, 7.6895e-04, 5.9885e-04, 3.8542e-04, 3.5256e-04 H/Ω for the Brooks coil and 7.6636e-04, 6.5807e-04, 7.6774e-04, 5.8848e-04, 3.1220e-04 H/Ω for the customised coil. Restricting the optimisation algorithm to the Brooks coil layout results in approximately 20% lower sensitivity than the use of customised shapes. Both designs have the following features: LT1028 pre-amplifier^12^, *f*_*0*_ = 10 kHz and *bw* = 19000 Hz.

To evaluate the benefit of using a tuned voltage-to-voltage configuration as opposed to the non-tuned current-to-voltage configuration, we perform tests based on the amplifier INA217 (*e*_*ni*_ = 3.5 nV/√Hz pink noise at 10 Hz + 1.3nV/√Hz white noise, *i*_*ni*_ = 3 pA /√Hz pink noise at 10 Hz + 0.8 pA/√Hz white noise, *e*_*on*_ = 90 nV/√Hz) and the amplifier LNA718A (*e*_*ni*_ = 2 nV/√Hz pink noise at 10 Hz + 0.7 nV/√Hz white noise, *i*_*ni*_ = 490 fA/√Hz pink noise at 10 Hz + 98 fA/√Hz, *e*_*on*_ = 13 nV/√Hz and *R*_*f*_ = 10 kΩ). In Fig. 6b, sensitivity is plotted against centre frequency for the four cases considered with *r*_*out*_ = 4.5 mm, *l*_*max*_ = 20 mm and *bw* = *f*_0_*1.9. For INA217, the non-tuned configuration is predicted to be more sensitive than the tuned one only when the centre frequency is below 21 Hz. For the LNA718A, the tuned configuration is over performs non-tuned configuration above 90 Hz.

**Figure 7:**
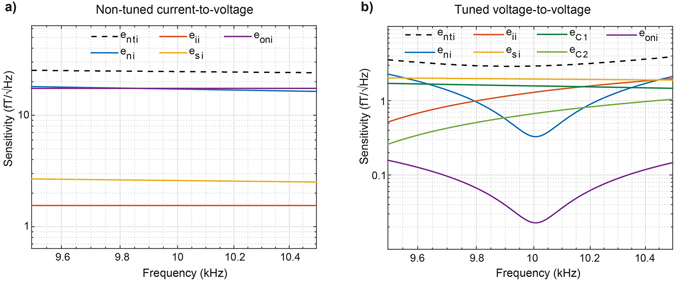
Assessment of various noise source contributions. Plots show the contribution of each noise source for the (**a**) non-tuned current-to-voltage and (**b**) tuned voltage-to-voltage configurations. These plots provide a visualisation of how various noise sources contribute in a frequency band of interest.

We use the numerical simulation environment to explore the effect of different noise sources on achievable sensitivity. In Fig. 7 we show results for the non-tuned current-to-voltage and the tuned voltage-to-voltage configurations optimised to a centre frequency of 10 kHz (*bw* = 1 kHz, *R*_*out*_ = 45 mm, amplifier INA217, *l*_*max*_ = 20 mm). The dominant noise sources for the non-tuned configuration (Fig. 7a) are the output (*e*_*oni*_) and input (*e*_*ni*_) voltage noise of the amplifier. For the tuned coil (Fig. 7b), thermal noise of the coil is dominant (*e*_*Si*_), closely followed by the thermal noise of the tuning capacitor in series with the coil (*e*_*C1*_) and the equivalent voltage noise generated by the current noise of the amplifier (*e*_*ii*_). Input voltage noise of the amplifier (*e*_*ni*_) is dominant in frequencies far from the centre frequency.

## Discussion

We propose a more general model for the design of air-core magnetometers for both non-tuned current-to-voltage and tuned voltage-to-voltage pre-amplifier configurations. We demonstrate that tuning can offer higher sensitivity even on wideband applications. By considering a more flexible model, we can produce air-core magnetometer configurations with very high predicted sensitivities. In particular, the sensitivity predicted for our optimised design is greater than that thought to be achievable with the optimal Brooks coils. Although this coil geometry has been proposed because of its high inductance to resistance ratio, the results we report suggest that this ratio does not necessarily provide the highest sensitivity. We show that additional gains in sensitivity can be achieved with an optimisation process accounting for a larger number of decision variables.

Our experimental results used for validation are in close agreement with the predictions from our model. Accurate characterisation of air-core magnetometers requires accurate estimation of the equivalent resistance, inductance and *emf*. The lower accuracy with which parasitic capacitance can be estimated is negligible, provided frequencies of interest are well below the self-resonant frequency of the coil. The model used to evaluate the gain in field (V/T) is verified for both the non-tuned and tuned configurations, alongside a validation for the noise floor. The good agreement between simulations and experimental findings is not unexpected, since we employ prevailing numerical models. We provide results for frequencies below 100 kHz. However, here presented numerical models are valid up to the low MHz range. From few MHz on other tools, such as the finite element method, maybe better suited due to wavelength shortening at higher frequencies.

With regard to the optimal frequency for maximal sensitivity in non-tuned air-core magnetometers, it has previously been shown that the sensitivity in the linear region is proportional to the cut-off frequency^16^. Therefore, it follows that the optimal sensitivity for a given bandwidth is near the cut-off frequency, as shown in Fig. 4a. For the tuned voltage-to-voltage configuration, the optimal sensitivity appears at a frequency close to the tuning frequency as a consequence of resonance. However, an improvement in sensitivity can be gained by shifting the tuning frequency away from the centre frequency of the bandwidth (Fig. 4c). The non-tuned current-to-voltage design only outperforms the tuned voltage-to-voltage design at ultra-low frequencies (<100 Hz) in the configurations examined here (Fig. 6).

The classical approach of optimising the air-core magnetometer sensitivity by matching the equivalent resistance *R*_*match*_ to *e*_*n*_/*i*_*n*_ does not always apply^22^. This approach is invalid here for a number of reasons. The classical approach assumes that noise sources *i*_*ni*_, *e*_*ni*_ and *e*_*si*_ are the main contributors of the total noise and *emf* increases proportionally with coil impedance. In the non-tuned current-to-voltage configuration, minimum output voltage noise of the amplifier can be dominant depending on the feedback of the amplifier, and the *emf* follows a nonlinear relationship with the impedance of the coil. In the tuned voltage-to-voltage configuration thermal noise of the tuning and matching capacitors can be dominant at very low frequencies. Additionally, most noise sources, as well as the equivalent source impedance, are frequency dependent in both configurations. Consequently, coil and network are not optimised to provide the best sensitivity at one single frequency but the best sensitivity averaged over the frequency bandwidth of interest.

We have shown different possibilities to enhance the sensitivity, the outcome of which can be anticipated though plots of individual noise contributions. In the example of Fig. 7 these noise contributions indicate that the minimum output noise and the voltage noise of the amplifier determine the noise floor of the non-tuned current-to-voltage magnetometer. Other noise sources are at least a factor of two smaller, reducing the benefit of using a Litz wire (see Fig. 5b). Conversely, in the tuned voltage-to-voltage configuration it is possible to improve sensitivity through the use of Litz wire because the thermal noise from the coil is dominant. The quality of capacitors used in the design should be considered carefully as their thermal noise can be significant (see Fig. 7b).

The use of lossy capacitors in the model confers notable benefits. First, the simulations are more reliable, showing a 75–80% improvement in the accuracy with the prototypes built for this work. Second, the optimisation process is forced to converge to feasible capacitor values. Third, the simulation environment can, to some extent, compensate for undesirable effects introduced by low quality capacitors via changes to the design.

Our results suggest a potential enhancement in air-core magnetometer sensitivity via exploitation of coil self-resonance in the tuned voltage-to-voltage configuration (see Fig. 5c). Any gains in sensitivity rely on being able to accurately predict the parasitic capacitance of the coil, which is problematic in the presence of densely packed loops. The parasitic capacitance is very susceptible to the manufacturing process and to any material imperfections. In effect, slightly different distances between loops generate different parasitic capacitances between neighbouring coils. This results in a broadening of the self-resonance peak and consequently reduces coil quality factor. We have avoided working with frequencies near the coil self-resonance to be able to build reproducible coils.

Our simulation environment could be modified to cater for design constraints not considered here. For example, linear sensitivity across frequencies may be desirable for some applications. For this case, any deviations in gain could be penalised in the optimisation process. Our results do, however, suggest that such a constraint will reduce sensitivity. In applications where the best noise performance is required, the gain could be linearised by adding a digital or analogue compensation step, such as the one used in^5^. In applications where a portable or lightweight solution is needed^7^, bounds on magnetometer weight could be set as well. Additionally, a fully capacitive T matching network may be beneficial if a very selective narrow band is required, noticing the additional losses from the extra capacitor. Networks incorporating inductors should be avoided due to the associated large losses, particularly at frequencies considered here. The coil layout could also be changed to account for cases where anisotropic wire positioning or non-rectangular profiles are desired. In such cases, particular modules of the numerical model, such as calculation of the AC resistance and parasitic capacitance of the coil, would need to be adapted on a case-by-case basis.

## Conclusions

We propose an optimisation method for the design of highly sensitive air-core magnetometers. Two popular amplification configurations are considered: non-tuned current-to-voltage and tuned voltage-to-voltage mode. We use a globally optimal method to maximise sensitivity by changing conductor diameter, spacing between conductors, number of conductor layers and loops per layer. Our findings suggest that the use of equations with fewer limiting assumptions and a greater number of decision variables yields air-core magnetometers which can significantly outperform existing designs such as the optimal Brooks coils.

The program used to generate the results is open source and it is publicly available via our website to help facilitate the design of high performance air-core magnetometers across a range of applications. The program can readily quantify the value added by the use of expensive electrical components such as high-end pre-amplifiers, high quality capacitors and Litz wires. Additionally, it can facilitate the design of customised, highly sensitive and relatively cheap air-core magnetometers to substitute more expensive and fragile technologies, such as SQUIDs, in applications like emerging low-cost fieldable ultra-low-field nuclear magnetic resonance systems^4, 23, 24^.

## References

[1] Dehmel, G. Magnetic field sensors: induction coil (search coil) sensors. *Sensors—A Comprehensive Survey*, 205–253, doi:10.1002/9783527620166.ch6 (1989).

[2] Tumanski S (2007). Induction coil sensors—A review. Measurement Science and Technology.

[3] Zakaria Z (2012). Advancements in transmitters and sensors for biological tissue imaging in magnetic induction tomography. Sensors.

[4] Matlashov AN (2011). SQUIDs vs. induction coils for ultra-low field nuclear magnetic resonance: experimental and simulation comparison. Applied Superconductivity, IEEE Transactions on.

[5] Estola K-P, Malmivuo J (1982). Air-core induction-coil magnetometer design. Journal of Physics E: Scientific Instruments.

[6] Grosz A, Paperno E (2012). Analytical optimization of low-frequency search coil magnetometers. IEEE Sensors Journal.

[7] Lukoschus DG (1979). Optimization theory for induction-coil magnetometers at higher frequencies. IEEE Transactions on geoscience electronics.

[8] Nourmohammadi A, Asteraki MH, Feiz SMH, Habibi M (2015). A Generalized Study of Coil-Core-Aspect Ratio Optimization for Noise Reduction and SNR Enhancement in Search Coil Magnetometers at Low Frequencies. IEEE Sensors Journal.

[9] Zelinka, I., Snasel, V. & Abraham, A. *Handbook of optimization: from classical to modern approach*. Vol. 38 (Springer Science & Business Media, 2012).

[10] Chen C, Liu F, Lin J, Wang Y (2015). Investigation and Optimization of the Performance of an Air-Coil Sensor with a Differential Structure Suited to Helicopter TEM Exploration. Sensors.

[11] Lin T (2014). High-sensitivity cooled coil system for nuclear magnetic resonance in kHz range. Review of scientific instruments.

[12] Tashiro K (2006). Optimal design of an air-core induction magnetometer for detecting low-frequency fields of less than 1 pT. Journal of the Magnetics Society of Japan.

[13] Timofeeva M, Allègre G, Robbes D, Flament S (2012). Differential search coils based magnetometers: conditioning, magnetic sensitivity, spatial resolution. Sensors & Transducers Journal.

[14] Yan B, Zhu W, Liu L, Liu K, Fang G (2013). An optimization method for induction magnetometer of 0.1 mHz to 1 kHz. IEEE Transactions on Magnetics.

[15] Yan B, Zhu W, Liu L, Liu K, Fang G (2015). Design of induction magnetometer receiving sensor for through-the-earth communications. IEEE Sensors Journal.

[16] Tashiro K, Inoue S-i, Wakiwaka H (2010). Sensitivity limits of a magnetometer with an air-core pickup coil. Sensors & Transducers.

[17] Savukov I, Seltzer S, Romalis M (2007). Detection of NMR signals with a radio-frequency atomic magnetometer. Journal of Magnetic Resonance.

[18] Martinez JL, Babic S, Akyel C (2014). On Evaluation of Inductance, DC Resistance, and Capacitance of Coaxial Inductors at Low Frequencies. Magnetics, IEEE Transactions on.

[19] Bowick, C. *RF circuit design*. (Newnes, 2011).

[20] Deep K, Singh KP, Kansal ML, Mohan C (2009). A real coded genetic algorithm for solving integer and mixed integer optimization problems. Applied Mathematics and Computation.

[21] Deb K (2000). An efficient constraint handling method for genetic algorithms. Computer methods in applied mechanics and engineering.

[22] Netzer Y (1981). The design of low-noise amplifiers. Proceedings of the IEEE.

[23] Sarracanie M (2015). Low-Cost High-Performance MRI. Scientific reports.

[24] Vogel MW, Giorni A, Vegh V, Pellicer-Guridi R, Reutens DC (2016). Rotatable Small Permanent Magnet Array for Ultra-Low Field Nuclear Magnetic Resonance Instrumentation: A Concept Study. PloS one.

